# Social Relationships and Depression: Ten-Year Follow-Up from a Nationally Representative Study

**DOI:** 10.1371/journal.pone.0062396

**Published:** 2013-04-30

**Authors:** Alan R. Teo, HwaJung Choi, Marcia Valenstein

**Affiliations:** 1 Department of Psychiatry, University of Michigan, Ann Arbor, Michigan, United States of America; 2 Department of Internal Medicine, University of Michigan, Ann Arbor, Michigan, United States of America; 3 Department of Veterans Affairs, HSR&D Center for Clinical Management Research Ann Arbor, Michigan, United States of America; University of Pennsylvania, United States of America

## Abstract

**Background:**

Social network characteristics have long been associated with mental health, but their longitudinal impact on depression is less known. We determined whether quality of social relationships and social isolation predicts the development of depression.

**Methods:**

The sample consisted of a cohort of 4,642 American adults age 25–75 who completed surveys at baseline in 1995–1996 and at ten-year follow-up. Quality of relationships was assessed with non-overlapping scales of social support and social strain and a summary measure of relationship quality. Social isolation was measured by presence of a partner and reported frequency of social contact. The primary outcome was past year major depressive episode at ten-year follow-up. Multivariable logistic regression was conducted, adjusting for the presence of potential confounders.

**Results:**

Risk of depression was significantly greater among those with baseline social strain (OR, 1.99; 95% CI, 1.47–2.70), lack of social support (OR, 1.79; 95% CI, 1.37–2.35), and poor overall relationship quality (OR 2.60; 95% CI, 1.84–3.69). Those with the lowest overall quality of social relationships had more than double the risk of depression (14.0%; 95% CI, 12.0–16.0; p<.001) than those with the highest quality (6.7%; 95% CI, 5.3–8.1; p<.001). Poor quality of relationship with spouse/partner and family each independently increased risk of depression. Social isolation did not predict future depression, nor did it moderate the effect of relationship quality.

**Conclusions:**

Quality of social relationships is a major risk factor for major depression. Depression interventions should consider targeting individuals with low quality of social relationships.

## Introduction

Major depressive disorder (MDD) is an illness of major clinical and public health significance. Lifetime prevalence of MDD in the United States is estimated at 16%, and the vast majority of episodes are characterized by at least moderate clinical severity and role impairment. [1] Also common in those with medical illnesses, depression increases risk for and worsens outcomes among those with coronary artery disease, [2] stroke, [3] and cancer. [4] Worldwide, major depressive disorder is a leading source of morbidity, and by 2030 it is projected to be the number one contributor to the global burden of disease. [5].

The association between mental health and social relationships has long been of interest. On a conceptual level, social relationships may influence mental health outcomes through multiple mechanisms including influence on health-related behaviors, engagement in social activities, transfer and exchange of social support, and access to material resources. [6], [7] On an empirical level, social isolation and negative social interactions are associated with depression [8], [9] and suicide. [10] Prospective community studies have found perceived marital dissatisfaction and negative marital quality at baseline are risk factors for an incident major depressive episode. [11], [12] Meta-analyses have shown that interventions addressing social relationships, including couples therapy and peer support may be effective in reducing depressive symptoms. [13], [14].

Still, the state of research on social relationships and mental health leaves a number of crucial issues unresolved. Most studies use cross-sectional data, [8], [9], [15] leaving investigators unable to determine the direction of causality between social relationships and mental health. Of the available longitudinal studies, many have focused on clinical populations [16] or been geographically restricted with modest sample sizes, [17] limiting generalization to the broader community. Finally, differing dimensions of social relationships are often not examined within the same study, including questions regarding with whom, how often, and in what way do people interact with others. This prevents direct comparisons of the relative importance of specific features of social relationships on depression.

This study assessed evidence for association of social relationships with depression in a prospective cohort. Our primary goal was to assess in a community population the role of both qualitative and quantitative aspects of social relationships in the subsequent development of major depression. We hypothesized that both poor quality of social relationships and social isolation would increase the risk of major depression ten years later. We also conducted secondary analyses to explore: 1) the relative impact of negative versus positive aspects of social relationships (strain versus support); 2) the relative impact of the quality of relationships with spouse, family, and friends; and 3) interactions between relationship quality and social isolation on the subsequent development of major depression.

## Methods

### Sample

Participants were from the Midlife in the United States (MIDUS) survey, a national longitudinal cohort study focused on the role of behavioral, psychological, and social factors in understanding physical and mental health. The sample was comprised of individuals selected by random digit dialing (RDD), with several geographical oversamples, siblings of individuals from the RDD sample, and a national RDD sample of twins. All eligible participants were non-institutionalized, English-speaking adults age 25–75 at baseline. The baseline assessment, Wave 1, occurred in 1995–1996, and a follow-up assessment, Wave 2, was conducted in 2004–2006. The full survey included a telephone interview, administered by trained lay interviewers, and a written questionnaire.

For our analyses, we included individuals who participated in both waves. After adjusting for mortality, the overall response rate of those in Wave 2 (n = 4,954) was 75%. [18] Those who continued study participation into Wave 2 tended to endorse more positive health-related variables, [18] a common phenomenon in longitudinal surveys. We further restricted our sample to those who had a valid response to the primary outcome variable (past-year major depressive episode at Wave 2) and were not missing all items that composed the primary predictor variable of relationship quality (n = 4,642). Primary analyses were conducted with those participants who had a spouse or partner at baseline (n = 3,500) with additional analyses additionally including the unpartnered (n = 4,642). Sample sizes for particular analyses vary due to differences in the number of evaluable responses. Detailed information on the MIDUS sample and study methods are available online (http://www.midus.wisc.edu/) and in print. [19].

### Measures

#### Past year major depression

Past-year major depressive episode, assessed at ten-year follow-up, was the primary outcome variable in this study. The diagnosis of major depressive episode was based on the Composite International Diagnostic Interview Short Form (CIDI-SF). For major depressive episodes, the CIDI-SF has a sensitivity of 89.6%, specificity of 93.9%, and overall agreement of 93.2% when compared to the full CIDI in a sample similar to this study. [20] Epidemiological studies employing the full CIDI have yielded prevalence rates of major depression similar to those in studies using clinical interviews such as the Structured Clinical Interview for DSM-III-R. [21] Numerous trials have documented good test-retest reliability and clinical validity of the CIDI-SF. [22], [23].

#### Quality of social relationships

In our primary analyses, we assessed the overall baseline quality of social relationships, including items measuring social support and strain. In secondary analyses, we used measures of the negative aspects of relationships (social strain) and the positive aspects of relationships (social support) separately. In additional secondary analyses, we assessed overall relationship quality for three different types of social relationships: spouse or partner, family members (excluding spouse/partner), and friends.

Positive, or supportive, aspects of social relations were based on a four-item scale about participants’ spouse or partner (Cronbach’s α = .86), family (Cronbach’s α = .83), and friends (Cronbach’s α = .88). [9], [24] Scale items were: “How much does your spouse or partner really care about you?”; “How much does he or she understand the way you feel about things?”; “How much can you rely on him or her for help if you have a serious problem?”; and “How much can you open up to him or her if you need to talk about your worries?” “To measure the combined effects of these three types of social relationships–spouse/partner, family, and friends–we constructed a composite scale of all items on social support (Cronbach’s α = .84). Four response options were available: 1 = ”a lot,” 2 = ”some” 3 = ”a little,” and 4 = ”not at all.” The scale score was the mean of individual items. The score range was from one to four, with a higher score indicating *less* social support.

Negative, or straining, aspects to social relations also consisted of four items about participants’ spouse or partner (Cronbach’s α = .80), family (Cronbach’s α = .78), and friends (Cronbach’s α = .79). 9,24 Scale items were: “How often does your spouse or partner make too many demands on you?”; “How often does he or she criticize you?”; “How often does he or she let you down when you are counting on him or her?”; and “How often does he or she get on your nerves?” To measure the combined effects of relationships with spouse/partner, family, and friends, we constructed a composite scale of all items on social strain (Cronbach’s α = .83). Response options and score range paralleled that of the positive interactions scale. Responses were reverse-coded, with a higher score indicating *more* social strain.

To measure overall relationship quality for the three different types of social relationships (spouse or partner, family members, and friends), we constructed composite, eight-item scales that combined the positive and negative dimensions of social relationships. Reliability was again good (Cronbach’s α for spouse or partner = .87; friend = .77; family = .82). Lastly, we constructed a composite scale of overall quality for all three types of social relationships–spouse/partner, family, and friends (Cronbach’s α = .87). On these four-point scales, a higher score indicates *lower* overall quality. Scale scores were the mean of all items.

#### Social isolation

Social isolation was assessed by determining at baseline whether someone lived with a marital or romantic partner and the frequency of their contact with non-cohabitating family, friends, and neighbors. Participants were asked, “Are you currently living with someone in a steady, marriage-like relationship?” They were also asked: 1) “How often are you in contact with any members of your family, that is, any of your brothers, sisters, parents, or children who do not live with you, including visits, phone calls, letters, or electronic mail messages?”; 2) “How often are you in contact with any of your friends – including visits, phone calls, letters, or electronic mail messages?”; and 3) “How often do you have a real conversation or get together socially with any of your neighbors?” For the first two items, eight response options were available, ranging from 1 = “several times a day” to 8 = “never or hardly ever.” For the item about contact with neighbors, the six response options varied from 1 = “almost every day” to 6 = “never or hardly ever.” In all cases, a higher score indicates *more* social isolation.

#### Covariates

Covariates were selected for inclusion based on their known association with depression or social relationships and all variables were assessed at baseline. Similar to recent studies, [17] we included sex, age, ethnicity, education level, household income, physical and mental health, major depressive disorder, generalized anxiety disorder, and alcohol misuse. Physical health was assessed using the single item, “In general, would you say your physical health is excellent, very good, good, fair, or poor?” Mental and emotional health was assessed similarly. Major depressive disorder and generalized anxiety disorder diagnoses were based on the CIDI-SF scales, which determined past 12-month prevalence. Alcohol misuse was assessed with a five-item alcohol screening test (e.g., “Did you ever, during the past 12 months, have such a strong desire or urge to use alcohol that you could not resist it or could not think of anything else?”), and if they answered “yes” to one or more items they were classified as having alcohol misuse. Variables were dichotomized when distribution of the data precluded analysis as continuous variables. All covariates were analyzed at baseline.

### Data Analyses

In our primary analyses, multivariable logistic regression was used to examine the association between the overall quality of social relationships, social isolation, and subsequent depression. A dichotomous measure of major depression was used because of sample skewness and the potential increase in clinical relevance (diagnosis rather than symptoms). Results are presented as odds ratios (ORs) with 95% confidence intervals (CIs). The regression models were adjusted for the effects of the covariates described above. We provide predicted probabilities of depression given differing levels of quality in relationships by evaluating at individual level risk values. Because our sample included a subpopulation of siblings and twins, we obtained robust standard errors by clustering error structure at the household level. We also conducted analyses with only the population-based (i.e., RDD) sample and found effect sizes similar to those reported herein. However, due to substantially reduced power, many of the results did not reach statistical significance in this reduced sample. Specification checks suggested the logit function was a linear combination of the predictors. Regression diagnostics indicated good discrimination and calibration of our model. We conducted an a priori power estimate, assuming a total sample size of 4,900 and 450 participants with social isolation or poor quality social relationships. With these parameters, we estimated a power of 78% to detect a 50% increase in depression (from 8% to 12%) and over 99% power to detect a doubling (from 8% to 16%).

Sample data were weighted using Current Population Survey (CPS) data derived from the Census Bureau to ensure that the sample was nationally representative in terms of age and gender distributions in 2005. As no sampling was conducted for Wave 2, only population-based adjustments were used. Details on the construction of the post-stratification weights are available in the supporting information **(Text S1 and Tables S1, S2, S3)**. All analyses reported herein are based on the weighted data. In addition to reflecting true household clusters, standardized errors also reflect variance in weights. [25].

We also conducted four sensitivity analyses. First, to account for missing responses, we employed multiple imputation using switching regression, an iterative multivariable regression technique. We used internal imputation for missing responses in individual items in the scales of social relationship quality (<3% missing) and covariates (<5% missing). There were no notable differences between results from the imputed and non-imputed datasets, and here we report results from the latter. Second, for analyses on participants with a spouse or partner, we added an additional covariate for change in partnership status to the multivariable models, which produced very similar effect sizes and no significant differences. Third, we repeated all analyses using unweighted data, with no significant differences in results detected. Fourth, we conducted analyses on the subsample of participants without major depression at baseline and report relevant results below.

Significance level for all tests was set at p<.05 (95% CI excluding one) and tests were two-tailed. Data were analyzed using Stata version 12 (Stata Corp.).

## Results

### Characteristic of Participants

**Table 1** provides baseline characteristics of the 4,642 study participants. Women comprised just slightly more than half of the sample and 92% were white. Five hundred fifty-nine participants (12.3%) had a major depressive episode at baseline. The mean score on the overall quality of social relationships was 1.82. Participants more commonly reported strain rather than lack of support in their social relationships. The majority did not have markers of social isolation.

**Table 1:** Baseline characteristics of the study sample (n = 4,642 unless otherwise noted).

Characteristic	n	% (weighted)
*Demographic*		
Female	2500	52.5
Age category		
25–34 years	881	28.9
35–44 years	1238	27.3
45–54 years	1191	19.9
55–64 years	851	12.3
65–75 years	481	11.6
Ethnicity (n = 4,581)		
White	4261	92.8
Black	177	3.7
Other	143	3.4
*Socioeconomic*		
Education (n = 4,634)		
Some grade school to some high school	324	6.5
Graduated high school/GED	1275	26.8
Some college	1406	30.6
Graduated college or higher	1629	36.1
Household income, dollars (n = 4,536)		
0–25,000	798	17.9
25,001–50,000	1170	26.2
50,001–100,000	1435	32.0
100,001+	1133	24.0
*Health Status*		
Very good or excellent overall physical health (n = 4,635)	2608	57.6
Very good or excellent overall mental health (n = 4,639)	2946	64.6
Major depressive episode	559	12.3
Generalized anxiety disorder	110	2.3
Alcohol misuse (n = 4,571)	625	15.5
		
	**Mean (weighted)**	**SD (weighted)**
*Quality of Social Relationships* a		
Overall poor quality with spouse/partner, family, and friends (n = )	1.81	0.36
Social strain with spouse/partner (n = 3,538)	2.22	0.60
Social strain with family (n = 4,555)	2.10	0.60
Social strain with friends (n = 4,600)	1.94	0.50
Lack of social support from spouse/partner (n = 3,545)	1.39	0.54
Lack of social support from family (n = 4,594)	1.55	0.60
Lack of social support from friends (n = 4,609)	1.76	0.65
*Social Isolation* b		
Lack of social contact with family (n = 4,574)	3.15	1.49
Lack of social contact with friends (n = 4,606)	3.36	1.66
Lack of social contact with neighbors (n = 4,624)	3.94	1.57
	**n**	**% (weighted)**
Unmarried or unpartnered	1142	25.1

a Overall poor quality, social strain, and lack of social support were measured on a four-point scale, with a higher score indicating poorer quality, more strain, and less support, respectively.

b Lack of social contact was measured on an eight-point scale for family and friends and a six-point scale for neighbors, with a higher score indicating more isolation.

### Relationship between Quality of Social Relationships and Depression

We first examined whether the overall of quality of social relationships at baseline predicted occurrence of major depressive episodes at follow-up. Across all types of social relationships, poor quality in core relationships was associated with a significantly higher risk of depression (adjusted odds ratio [AOR], 2.65; 95% CI, 1.86–3.76), even after accounting for the predictive power of baseline major depression and other covariates. In secondary analyses including all types of social relationships, both strain (AOR, 2.03; 95% CI, 1.49–2.76) and lack of support (AOR, 1.79; 95% CI, 1.36–2.36) were also associated with increased risk of depression (**Table 2**). In sensitivity analysis among partnered participants who did not have a major depressive episode at baseline (n = 3,154), results were still highly significant (for overall poor quality: AOR, 2.54; 95% CI, 1.71–3.76; for social strain: AOR, 2.33; 95% CI, 1.64–3.29; for lack of social support: AOR, 1.57; 95% CI, 1.14–2.16).

**Table 2:** Risk of major depressive episode at ten-year follow-up as a function of overall quality of social relationships, social support, and social strain.

	Participants with spouse/partner at baseline			All participants at baseline		
Component of relationship quality at baseline	n	Adjusted OR^a^ (95% CI)	Robust SE	N	Adjusted OR^a^ (95% CI)	Robust SE
**Overall poor quality**	3287	2.60 (1.84–3.69)	0.46	4278	1.94 (1.46–2.59)	0.29
**Social strain**	3326	1.99 (1.47–2.70)	0.31	4312	1.51 (1.18–1.92)	0.19
**Lack of social support**	3357	1.79 (1.37–2.35)	0.25	4345	1.46 (1.19–1.80)	0.16

a Adjusted for the following variables at baseline: major depression, age, ethnicity, sex, household income, education level, generalized anxiety disorder, alcohol misuse, overall physical health, and overall mental health. Social strain, lack of social support, and overall poor quality in relationships were rated on a four-point scale and each was in a separate multivariable model. For analyses with participants with a spouse/partner, scales of social relationships quality are a composite of relationships with spouse/partner, family, and friends; for analyses of all participants, scales contain just the latter two relationships.

In secondary analyses, we assessed relationships only with family and friends, including all participants, even those without a partner. Again we found that overall poor quality in relationships was a significant predictor of depression. Additionally, sensitivity analysis among participants who did not have a major depressive episode at baseline (n = 4,083) also showed significant results.

### Relationship between Quality of Different Types of Social Relationships and Depression

The type of social relationship also affected depression risk (**Table 3**). In this secondary analysis, poor overall quality of relationship with one’s spouse/partner (AOR, 1.47; 95% CI, 1.16–1.87) and family members (AOR, 1.45; 95% CI, 1.10–1.90) significantly and independently increased risk of depression. In contrast, the overall quality of relationships with friends did not independently predict subsequent depression (AOR, 1.21; 95% CI,.84–1.72).

**Table 3:** Risk of major depressive episode at ten-year follow-up as a function of type of social relationship with overall poor quality at baseline.

	Participants with spouse/partner at baseline			All participants at baseline		
Type of relationship	n	Adjusted OR^a^ (95% CI)	Robust SE	n	Adjusted OR^a^ (95% CI)	Robust SE
**Spouse/partner**	3287	1.43 (1.13–1.82)	0.17	n/a	n/a	n/a
**Family**	3287	1.47 (1.12–1.93)	0.20	4278	1.54 (1.21–1.95)	0.19
**Friends**	3287	1.20 (0.84–1.72)	0.22	4278	1.23 (0.92–1.65)	0.18

a Adjusted for the following variables at baseline: major depression, age, ethnicity, sex, household income, education level, generalized anxiety disorder, alcohol misuse, overall physical health, and overall mental health. Quality in relationships was rated on a four-point scale. Spouse/partner, family, and friends were included in the same multivariable model for analyses of participants with a spouse/partner and the just the latter two for analyses with all participants.

Similarly, in sensitivity analysis where participants without a spouse or partner were included, poor quality of relationships with family members but not friends significantly increased risk of depression. In the subset of participants without major depression at baseline (n = 3,133), poor quality of relationship with one’s spouse/partner (AOR, 1.46; 95% CI, 1.10–1.93) significantly and independently increased risk of depression, but results were not significant for family members (AOR, 1.27; 95% CI,.90–1.79) or friends (AOR, 1.43; 95% CI,.95–2.16).

### Predicted Probability of Depression Depending on Quality of Social Relationships

**Figure 1:**
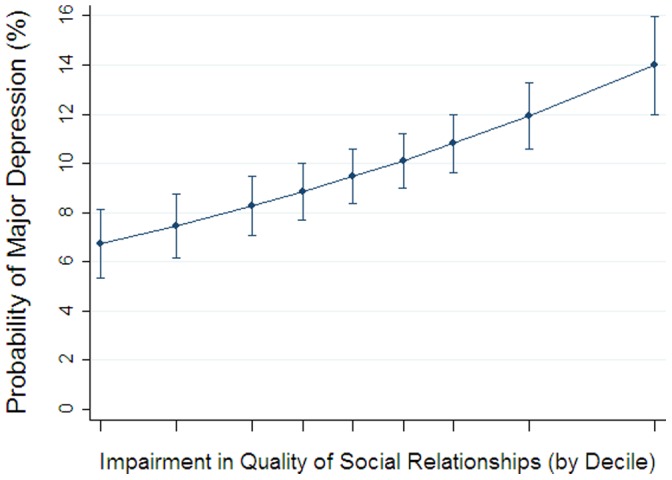
Predicted probability of depression. Impairment in quality of social relationships was rated on a four-point scale, with a higher score indicating *more impairment* in overall quality in relationships with spouse/partner, family, and friends. Data are weighted to adjust results to more closely match characteristics of the general U.S. population.

Using our multivariable logistic regression model from our primary analyses, we estimated the composite effect of the overall quality of participants’ social relationships with family, friends, and spouse/partner at baseline on likelihood of developing depression ten years later. Those with the highest quality social relationships (top decile) had just a 6.7% chance of major depression (95% CI, 5.3–8.1; p<.001), whereas those with the lowest quality (bottom decile) had a 14.0% chance (95% CI, 12.0–16.0; p<.001). **Figure 1** presents the estimated risk of depression, illustrating a “dose-dependent” effect of impairment in quality of social relationship on risk for major depression.

### Relationship between Social Isolation and Depression

Data on frequency of social contact were available for relationships with family, friends, and neighbors. In the model, lack of contact with each type of social relationship at baseline was included as a separate predictor variable. Results indicated that lack of social contact, whether with family (AOR, 1.02; 95% CI,.95–1.09), friends (AOR, 1.00; 95% CI,.94–1.06), or neighbors (AOR, 1.03; 95% CI,.96–1.11), did not predict risk of depression. Furthermore, being married (AOR, 1.12; 95% CI,.88–1.44) or having a romantic partner (AOR, 1.10; 95% CI,.85–1.42) at baseline was not associated with future depression.

### Interactive Effects of Social Isolation and Quality of Social Relationships

We were interested in whether frequency of social contact acted as a moderator on the impact of overall quality of social relationships on depression. In the case of both family (AOR, 2.72; 95% CI,.67–11.1) and friends (AOR,.89; 95% CI,.42–1.90), lack of contact did not significantly interact with quality of relationship. We also examined whether there was an interaction between quality of social relationships variables and depression at baseline. In models for overall quality of relationships with family, friends, and spouse/partner, there were no interactions found.

## Discussion

### Importance of Considering of Social Relationships in Mental Health

This observational study of a large nationally representative community cohort demonstrates that the quality of social relationships–even after accounting for baseline depression and other important potential confounders–predicts future depression. Results were similar when analyses were restricted to those participants without major depression at baseline, which suggests that the predictive power of social relationship quality is not explained by depression’s influence on self-report of one’s social relationships. Remarkably, this effect appears to be very durable, predicting development of clinically significant depression ten years later. This long-term effect extends earlier studies that have demonstrated that negative social interactions predict negative affect three months later [26] and that perceived social support predicts depression outcome up to three years later. [16].

Both the negative and positive qualities of the relationship were independently predictive of depression, with the effect of negative aspects being modestly stronger. Prior cross-sectional studies have pointed out the detrimental effect of negative social interactions, [9], [27] though the bulk of the literature focuses on the positive aspects of social support. We reinforce here the importance of social strain–not just support–when considering mental health. In addition, our data suggest the value of evaluating overall quality of social relationships as a risk factor for depression.

When assessing the quality of social relationships, it also appears worthwhile to consider which type of social relationship has strain or lacks support. Specifically, our data suggests that having problems especially with one’s spouse and less so with other family members–but not friends–each exert an independent effect on depression risk. This extends and corroborates earlier cross-sectional research that showed not getting along with one’s spouse was related to more psychiatric disorders than not getting along with relatives or friends. [15] Health care providers should remember that patients’ relationships with their loved ones likely play a central role in their medical care, [28] whereas weaker social ties tend to impact other aspects of life. [29].

We did not find support for our hypothesis, that frequency of social contact would impact the likelihood of future depression. Moreover, our analyses on interactions indicated that even if participants had little contact with their family or friends, this social isolation did not moderate the effect of quality of social relationships on subsequent depression risk. Though objective social isolation–social network characteristics such as few or infrequently contacted social relationships–has been associated with mental illness, the association appears to be weak. [8] Our results thus support the argument that *subjective* components of social relationships are more critical to one’s health than *objective* characteristics of one’s social network. This is a robust finding that has been observed not only for mental health outcomes [17], [30] but also for cardiovascular disease, [31], disability, [32] and mortality. [33].

We estimate that one in seven adults who have social relationships in the bottom decile of relationship quality will develop major depression years later, whereas, just one in fifteen of those with the highest quality of social relationships will develop depression, suggesting substantial benefits at the population-level if people can learn how to improve the quality of their social relationships. This sort of effect size is clinically meaningful and can be illustrated by comparing it to other research on risk factors for major health outcomes. Specifically, data from the famous Framingham study indicate the following: to achieve a similar reduction in ten-year risk of a myocardial infarction from 15% to 7%, a 60-year-old non-smoker with a systolic blood pressure of 120 mm not on pharmacotherapy for hypertension would have to have his total cholesterol decrease from 300 mg/dL to 200 mg/dL and HDL cholesterol increase from 40 mg/dL to 60 mg/dL. [34].

### Implications for Interventions

Social relationships may be a critical target for public health officials and clinicians alike: the magnitude of effect is sizable, the prevalence of poor quality social relationships is high, and the population-level morbidity of major depressive disorder is among the highest of any condition. Asking patients about their subjective perceptions of their social relationships should be a priority. Including questions in the clinical encounter about, for instance, how much others care and understand the patient, as well as how much others’ criticize and let the patient down should be considered evidence-based, much like inquiring about past depressive episodes.

Furthermore, treatments that focus on ameliorating one’s social relationships may be a particularly helpful for depression. One treatment strategy is via individual psychotherapy that modifies patients’ emotional or cognitive perceptions about their interpersonal relationships. Two time-limited, evidence-based psychotherapies, cognitive behavioral therapy and interpersonal therapy, are effective treatments for depression and can be used to directly target problems with social relationships. Indeed, a large meta-analysis found cognitive therapy approaches to be the most efficacious for lonely subjects. [35] Interpersonal psychotherapy–which can focus on areas such as conflicts in one’s social relationships–is efficacious as both acute and maintenance treatment for depression, [36] though dissemination remains low. [37] Public health officials and policy makers should consider supporting broader investigation and uptake of these treatments. Given our results showing the strength of spouse/partner relationship quality as a predictor for depression, the broader use of couples therapy might also be considered. Couples therapy–in which both partners attend sessions led by a therapist with the aim of promoting supportive aspects of their relationship and reducing patterns of negative interaction–has been shown to be effective for mild to moderate depression. [14] It may also be worthwhile investigating its potential as a preventive treatment for major depression.

### Limitations

Several limitations of this study deserve mention. First, confounding by unmeasured variables is a threat in any observational study, perhaps even more so in studies like this that examines social determinants of health. However, we have utilized advanced statistical methods to address potential confounding and offer conservative estimates of confidence intervals. Third, though this study was longitudinal it only contains two timepoints for analysis. As a consequence, it is possible that in the long interim interval participants’ quality of social relationships varied. However, prior research has indicated that negative social interactions, at least in older adults, is quite stable over years. [38] Fourth, these analyses lack the granularity in data to distinguish between incident and recurrent major depression, though given the age of participants and the known epidemiology of depression, it is likely that many cases were recurrent. Therefore, we are unable to report whether social relationship quality is more or less of a predictor for first-episode or recurrent depression. Finally, several measures relied on participants’ recall of over the prior year, a lengthy period of time.

### Conclusion

In summary, this study suggests that social relationships may be an important area to target among adults at risk for clinical depression. The mantra that quality is more important than quantity appears true in the effect of social relationships on depression. The magnitude of effect of social relationship quality on risk for depression is comparable with the effect of well-established biological risk factors for cardiovascular disease. Interventions for individuals with low quality of social relationships, and therefore high risk for future major depressive episodes, merit more investigation and dissemination.

## Supporting Information

Table S1 Cross-Classification of Unweighted MIDUS Wave 2 Sample.
(DOCX)

Table S2 Cross-Classification of CPS Sample in 2005.
(DOCX)

Table S3 Post-stratification Weights Using Age Strata and Gender.
(DOCX)

Text S1 **Procedure for Creating Post-Stratification Weights for the Study Sample.** Step-by-step description of methods and supplemental tables for creating post-stratification weights for the study sample.
(DOCX)
