# Supplementary material for: Social Relationships and Depression: Ten-Year Follow-Up from a Nationally Representative Study
Source: PLoS One. 2013 Apr 30;8(4):e62396. doi: 10.1371/journal.pone.0062396 (PMC3640036; doi:10.1371/journal.pone.0062396)
Supplement: Table S1 — Cross-Classification of Unweighted MIDUS Wave 2 Sample. (DOCX) [file pone.0062396.s001.docx]

**Table S1: Cross-Classification of Unweighted MIDUS Wave 2 Sample (n=4,642)**

|  | GENDER | | | |
| --- | --- | --- | --- | --- |
|  | Male | | Female | |
| AGE | n | % | n | % |
| 33-44 | 428 | 9.2 | 568 | 12.2 |
| 45-54 | 618 | 13.3 | 642 | 13.8 |
| 55-64 | 557 | 12.0 | 601 | 12.9 |
| 65-74 | 351 | 7.6 | 466 | 10.0 |
| 75-84 | 188 | 4.0 | 223 | 4.8 |
