# Supplementary material for: Social Relationships and Depression: Ten-Year Follow-Up from a Nationally Representative Study
Source: PLoS One. 2013 Apr 30;8(4):e62396. doi: 10.1371/journal.pone.0062396 (PMC3640036; doi:10.1371/journal.pone.0062396)
Supplement: Table S2 — Cross-Classification of CPS Sample in 2005. (DOCX) [file pone.0062396.s002.docx]

**Table S2: Cross-Classification of CPS Sample in 2005 (n=158,554)**

|  | GENDER | | | |
| --- | --- | --- | --- | --- |
|  | Male | | Female | |
| AGE | n | % | n | % |
| 33-44 | 25721 | 16.2 | 26128 | 16.5 |
| 45-54 | 20555 | 13.0 | 21405 | 13.5 |
| 55-64 | 14051 | 8.9 | 15485 | 9.8 |
| 65-74 | 8466 | 5.3 | 9924 | 6.3 |
| 75-80* | 6681 | 4.2 | 10139 | 6.4 |

*CPS data are only available up to age 80, but our sample included a small number of participants over 80 (1.1% of sample); therefore, for MIDUS participants age 75-84, we assigned the weight based on CPS data for people age 75-80
