# Supplementary material for: Social Relationships and Depression: Ten-Year Follow-Up from a Nationally Representative Study
Source: PLoS One. 2013 Apr 30;8(4):e62396. doi: 10.1371/journal.pone.0062396 (PMC3640036; doi:10.1371/journal.pone.0062396)
Supplement: Table S3 — Post-stratification Weights Using Age Strata and Gender. (DOCX) [file pone.0062396.s003.docx]

**Table S3: Post-stratification Weights Using Age Strata and Gender**

|  | GENDER | |
| --- | --- | --- |
|  | Male | Female |
| AGE |  |  |
| 33-44 | 1.76 | 1.35 |
| 45-54 | 0.98 | 0.98 |
| 55-64 | 0.74 | 0.76 |
| 65-74 | 0.70 | 0.63 |
| 75-84 | 1.04 | 1.33 |
