# Supplementary material for: Social Relationships and Depression: Ten-Year Follow-Up from a Nationally Representative Study
Source: PLoS One. 2013 Apr 30;8(4):e62396. doi: 10.1371/journal.pone.0062396 (PMC3640036; doi:10.1371/journal.pone.0062396)
Supplement: Text S1 — Procedure for Creating Post-Stratification Weights for the Study Sample. Step-by-step description of methods and supplemental tables for creating post-stratification weights for the study sample. (DOCX) [file pone.0062396.s004.docx]

**Procedure for Creating Post-Stratification Weights for the Study Sample**

1. Select demographic variables to use for adjustment. In this case, age and gender were chosen.
2. Tabulate frequency and proportion of the study sample, separated into cross-classifications. Gender has two options and age was categorized into five strata, resulting in a total of 10 cross-classifications (**see Table S1**).
3. Tabulate national population data from the Current Population Survey (CPS) using the same cross-classifications (**see Table S2**).
4. Compute the new weights. Weight values will be a simple division of the population proportion by the sample proportion for all 10 of the cross-classifications (**see Table S3**).
